# Supplementary material for: Steam recovery from flue gas by organosilica membranes for simultaneous harvesting of water and energy
Source: Nat Commun. 2023 Nov 23;14:7641. doi: 10.1038/s41467-023-43546-y (PMC10665434; doi:10.1038/s41467-023-43546-y)
Supplement: Supplementary file 1 — Supplementary Information [file 41467_2023_43546_MOESM1_ESM.pdf]

Supplementary Information

Steam recovery from flue gas that enables the simultaneous harvesting of water and energy: proof-of-concept

Norihiro Moriyama,[1] Akihiro Takeyama,[2] Taichi Yamatoko,[2] Ken-ichi Sawamura,[3] Koji Gonoi,[3] Hiroki Nagasawa,[1] Masakoto Kanezashi,[1] and Toshinori Tsuru[1]

[1]Department of Chemical Engineering, Hiroshima University, 1-4-1 Kagami-yama, Higashi-Hiroshima 739-8527, Japan

[2]PLANTEC Inc., 1-6-17 Kyomachibori, Nishi-ku Osaka city 550-0003, Japan

[3]eSep Inc., Keihanna Open Innovation Center, 7-5-1 Seikadai, Seika-cho, Souraku-gun, Kyoto 619-0238, Japan

---

Corresponding author: Toshinori Tsuru (tsuru@hiroshima-u.ac.jp)

Keywords: Steam recovery; Flue gas; Organosilica membrane; Vapor permeation; Long-term stability; Incinerator plant

---

Inventory of Supporting Information

(Pages 3-6)

Supplementary Note 1: Comparing conventional waste incinerator plants with a proposed version

(Pages 7-9)

Supplementary Note 2: Vapor permeation (VP)

(Pages 10-20)

Supplementary Note 3: Simulation of the steam recovery system

Supplementary Note 3-1: Variations of steam recovery systems

Supplementary Note 3-2: Example of streams in membrane unit and heat exchanger

Supplementary Note 3-3: Example of steam recovery via membrane (for Case Ⅰ and Case Ⅱ)

Supplementary Note 3-4: Energy recovery via heat exchanger

Supplementary Note 3-5: Recovery and operational energy costs

(Page 21)

Supplementary Note 4: Competing technologies for steam recovery

(Page 22)

Supplementary Note 5: Membrane performance after long-term stability testing (laboratory-scale test)

(Pages 23-24)

Supplementary Note 6: Time course of steam recovery experiments

(Pages 25-26)

Supplementary Note 7: Downstream pressures in the bench-scale apparatus

(Page 27)

Supplementary Note 8: Cross-sectional SEM image of an organosilica membrane

(Pages 28)

Supplementary Note 9: Thermodynamic properties used to evaluate effective heat recovery

(Pages 29-31)

Supplementary References

Supplementary Note 1: Comparing conventional waste incinerator plants with a proposed version

Supplementary Figure 1 illustrates (a) a conventional waste incinerator plant that uses a fresh water supply, (b) a self-reliant plant with a heat exchanger, and (c) the proposed self-reliant plant with a steam recovery membrane unit. Conventional plants (case (a)) use as much as 74 t d^{-1} of fresh water to rapidly cool the stream from a combustion furnace to prevent the production of dioxins. The stream, which contains significant amounts of latent heat, is then released into the atmosphere from the stack. Another negative factor is that such a large amount of steam forms a steam condensate plume. The appearance of a steam condensate plume is a concern for the general public and often complicates the construction of incinerator plants in urban areas. This effect extends to all types of industrial plants, including power and chemical plants. Some plants re-heat the combustion gas stream simply to prevent the appearance of a steam condensate plume.

Please refer to Supplementary Table 1, which summarizes performances of (a) a conventional waste incinerator plant that uses a fresh water supply, (b) a self-reliant plant with a heat exchanger, and (c) the proposed self-reliant plant equipped with a steam recovery membrane unit. With the use of a heat exchanger (case (b)) or a steam recovery membrane unit (case (c)), steam contained in the stream after dust filtration can be recovered and reused, which amounts to a self-reliant waste incinerator plant without the need for a fresh water supply. This would be of great importance in any region with a shortage of water. In addition, both options b and c enable the simultaneous recovery of latent heat and water. However, the water recovered via a heat exchanger is of lower quality, containing contaminants such as HCl, SO_x and NO_x. Recycling this water leads to a concentration of impurities, promoting corrosion within the process pipes and equipment. In contrast, water collected by a steam recovery membrane unit is clean because the membrane prevents the permeation of such contaminants (see Fig. 3). Moreover, generally the heat transfer efficiency of heat exchangers diminishes significantly when non-condensable gases exist, resulting in large heat exchanger volumes (Please see Supplementary Figure 2). In the case of option (c), the downstream of the membrane consists of almost pure steam, which results in high heat transfer efficiency and less device volume compared with the use of a heat exchanger. Another point to be compared is the steam condensate plume from the stack. When a heat exchanger is used directly, the processed stream must be cooled. The stream from the heat exchanger becomes saturated with water vapor, reaching a dew point. This results in the appearance of a steam condensate plume from the stack. With the use of a steam recovery membrane unit, only the recovered stream (downstream of the membrane) must be cooled and the non-permeating (dehydrated) stream maintains its high temperature (150-200 °C). As a result, the humidity of the non-permeating stream should be 1-3%, which results in almost the complete absence of a steam condensate plume.

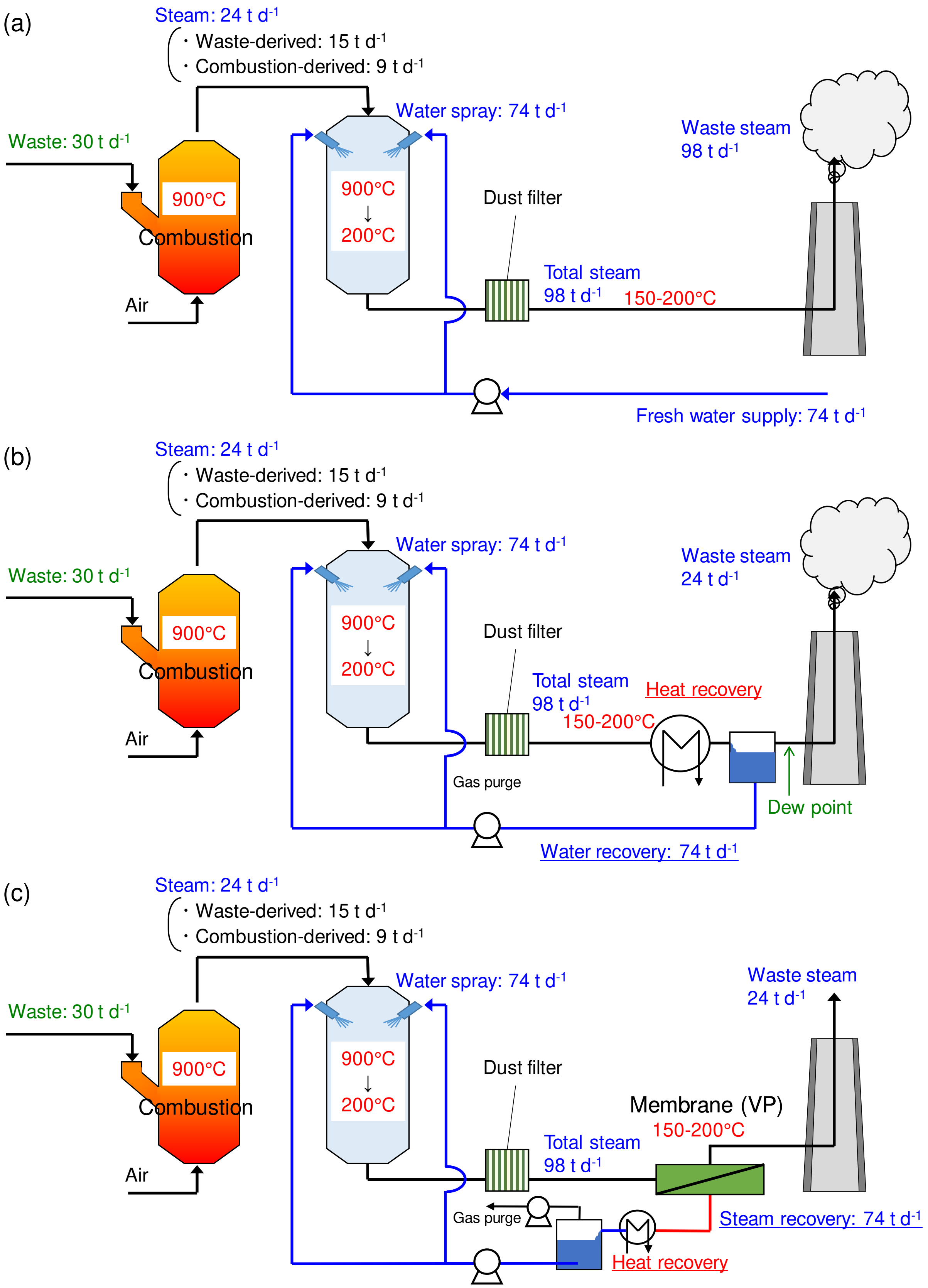

Supplementary Figure1 Water balance in waste incinerator plants with a waste capacity of 30 t d^{-1}: (a) A conventional plant using a fresh water supply; (b) A self-reliant plant using a heat exchanger; and, (c) The proposed self-reliant plant equipped with a steam recovery membrane unit.

Supplementary Table 1 Comparison of waste incinerator plants: (a) conventional, (b) with a heat exchanger, (c) with a steam recovery membrane unit

	(a) Conventional	(b) With heat exchanger	(c) With membrane
Water recovery	No	Yes	Yes
Quality of recovered water	-	Bad	Good
Heat recovery	No	Yes	Yes
Heat exchange efficiency	-	Low	High
Steam condensate plume	Much	Much	No or Little

The heat transport coefficient can be expressed as Eq. (S1).

$$Q' = UA(T_h - T_l) \qquad \mathrm{Eq.}(S1)$$

In Eq. (S1), Q', U, A, T_h, and T_l are the heat flow rate, overall heat transfer coefficient, area and temperatures of high and low temperature fluids, respectively.

Supplementary Figure 2 illustrates the temperature drops across the wall of a heat exchanger (a) without and (b) with a steam recovery membrane unit where Q', A and T_h are constant. In case (a), the collection of water vapor condensation on the surface and within the boundary layer leads to an increased concentration of non-condensable gases increases due to the selective condensation of steam in the processing gas-flow side. The temperature dramatically decreases across the concentrated gas layer because it prevents steam diffusion from the high-temperature side to the low-temperature side. On the other hand, in case of (b), the process stream that permeates organosilica membranes is almost pure steam, which prevents the formation of thick non-condensable gas layer. So, a bit of only a thin temperature boundary layer and a condensed water layer are created for heat-transfer resistance in the processing from the gas-flow side. Reportedly,[1] 1% of non-condensable gas reduces the heat transfer efficiency to only several-tens of % points compared with that of pure steam. Comparing Figs. (a) and (b), obviously T_l with a steam recovery membrane unit is higher than that without it. This indicates that the steam recovery membrane unit improves the overall heat transfer coefficient, U, in Eq. (S1).

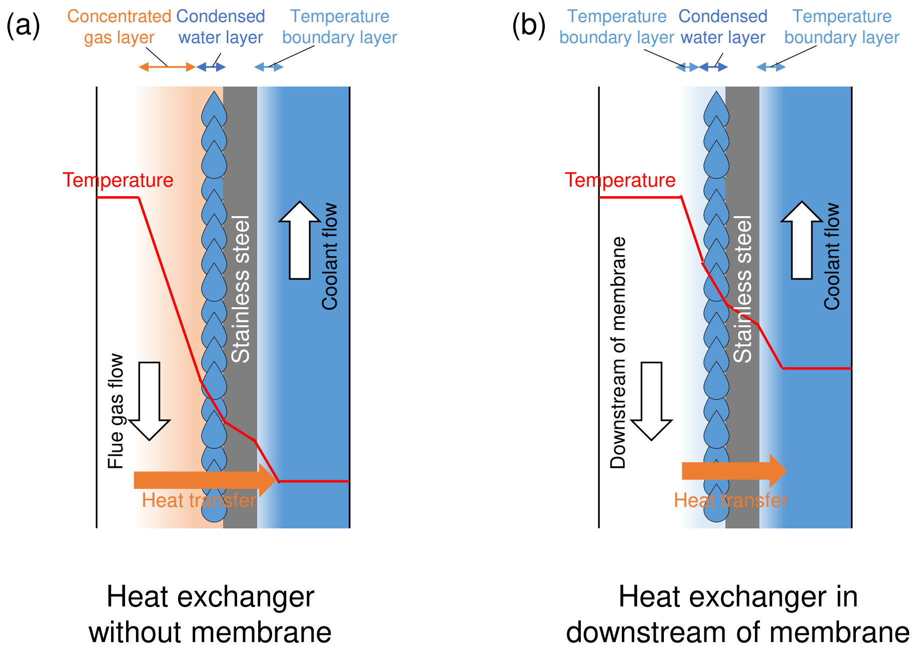

Supplementary Figure 2 Illustration of heat transfer in a heat exchanger (a) without (case (b) in Supplementary Figure 1) and (b) with a steam recovery membrane (case (c) in Supplementary Figure 1).

Supplementary Note 2: Vapor permeation (VP)

Permeation of condensable components such as water through molecular-selective membranes can be categorized into three modes according to a combination of the upstream and downstream phases. As schematically shown in Supplementary Figure 3, in vapor permeation, both the upstream and downstream are in the gas phase. The permeating flux of component-*i*, J_i [mol m^{-2} s^{-1}], can be expressed as Eq. (S2).

$$J_i = \Pi_i(p_{1,i} - p_{2,i}) \quad \text{Eq. (S2)}$$

In Eq. (S2), Π_i, $p_{1,i}$, and $p_{2,i}$, are the permeance (a constant related to membrane performance) and partial pressures in the upstream and downstream phases of component-*i*, respectively. Here, $p_{1,i} - p_{2,i}$, the partial pressure difference of component-*i,* is the driving force for permeation.

Since the driving force is given by the pressure difference, the upstream usually has higher pressure than the downstream.

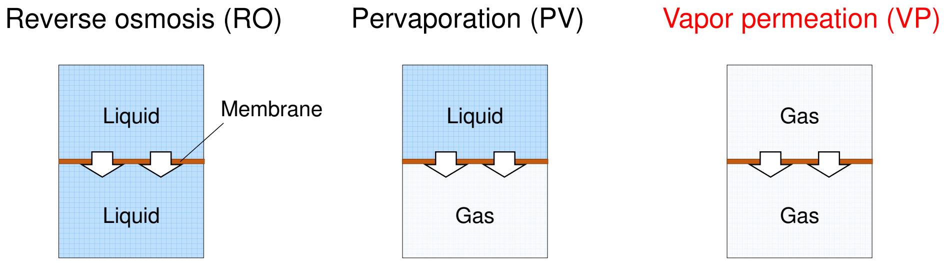

Supplementary Figure 3 Permeation modes of condensable components through molecular-selective membranes.

Supplementary Figure 4 schematically illustrates a membrane module with three flows: feed, retentate and permeate. “Feed” indicates a process stream that is fed to the membrane module for separation, which is followed by selective permeation of the water vapor via the membrane. The membrane-permeating and non-permeating component flows are referred to as the “permeate” (or downstream) and “retentate”, respectively, and also are referred to as downstream and upstream, respectively.

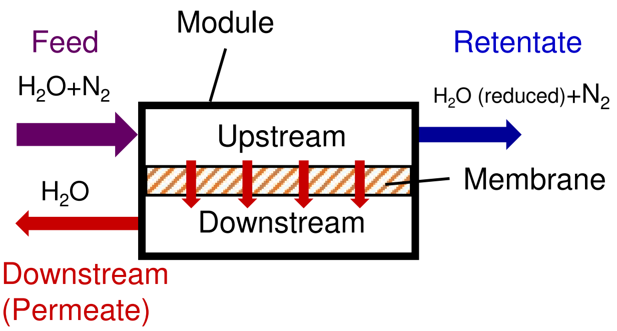

Supplementary Figure 4 A schematic membrane module that separates water vapor from the mixture of water vapor and nitrogen.

Separation of water vapor from gases has many important applications such as the drying of natural gas, humidity control of air, and the dehydration of compressed air. [2-5] In addition, since water vapor sometimes induces corrosion in chemical plants, dehumidification of process gases via VP can be an anticorrosion technology. [6] The feed gases are usually at low temperature with low water vapor pressure, so that downstream of the membrane is usually evacuated to promote permeation according to Eq. (S2).

Supplementary Table 2 summarizes VP membranes for water vapor/gases separation found in the literature. Organic membranes are mostly used for the dehumidification of air, fuel, and sampling gas. They are usually operated at temperatures below 100 °C, because of the limitations in thermal stability. Some thermally stable organic membranes such as perfluorosulfonic acid (PFSA) are applied to steam/gas separation at high temperature, but the permeance is low. [7] Under high temperature, inorganic membranes such as zeolite and silica have been thoroughly studied. Although inorganic membranes are thermally stable compared with polymeric membranes, the hydrothermal stability is still challenging. One of the most attractive applications is the removal of steam by-product in an equilibrium reaction such as in Fischer-Tropsch synthesis, which can improve the conversion beyond the equilibrium. [8,9] Steam/N_2 separation performance at high temperature (above 150 °C) is important for steam recovery, and has been evaluated in only a limited number of papers. A summary of steam/N_2 separation performance at temperatures ranging from 150 to 300 °C, as reported in the literature, can be found in SI-4. In this study, we fabricated organosilica membranes based on the developed structure of separation layer as well as the layered structure, and successfully confirmed excellent hydrothermal stability (~200°C, ~50 kPa-a H_2O) even under HCl (40-400ppm).

Supplementary Table 2 VP membranes for water vapor/gases separation found in the literature with their operating temperatures[3,5,7-21]

Material	Abbreviation	Material type	Temperature [°C]	Reference
Ethyl cellulose	EC	Organic	30	10
Cellulose acetate	CA	Organic	30	10
Natural rubber	NR	Organic	30	10
1000PEO56PBT44	PEO-PBT	Organic	20-80	10
Polyacrylonitrile	PAN	Organic	30	3, 10
Polyamide	PA	Organic	30	10
Polycarbonate	PC	Organic	30	10
Polydimethylsiloxane	PDMS	Organic	30	10
Polyethersulfone	PES	Organic	30	10
Polyethylene	PE	Organic	30	10
Polyimide	PI	Organic	30	10
Polyphenyleneoxide	PPO	Organic	30	10

Polypropylene	PP	Organic	30	10
Polystyrene	PS	Organic	30	10
Polysulfone	PSF	Organic	30-50	10, 16
Polyvinylalcohol	PVA	Organic	30	10
Polyvinylchloride	PVC	Organic	30	10
Sulfonated polyetheretherketon	SPEEK	Organic	30-70	10, 18
Sulfonated polyethersulfone	SPES	Organic	30	10
Perfluorosulfonic acid	PFSA	Organic	20-150	7, 19, 20
Graphene oxide	GO	Inorganic	22-43	17
Zeolite	LTA	Inorganic	25-400	5, 9
Zeolite	MFI	Inorganic	25-452	8, 9, 15,
Zeolite	MOR	Inorganic	25-452	8, 9, 15,
Zeolite	FAU	Inorganic	130-180	8
Zeolite	SOD	Inorganic	200-250	9
Alumina	Al_2O_3	Inorganic	250	9
Silica	SiO_2	Inorganic	250-500	9, 21
Organosilica	-	Hybrid	80-200	11-14

Supplementary Note 3: Simulation of the steam recovery system

Supplementary Note 3-1: Variations of steam recovery systems

Supplementary Figure 5 showcases two variations of the steam recovery system in a waste incinerator plant.

In Case I, the downstream of the membrane is cooled through a heat exchanger, subsequently liquifying the water which is then stored in a tank. The pump after the heat exchanger in this setup is used to purge non-condensable gases. Given that the downstream of the membrane mainly consists of pure steam with only a minimal amount of non-condensable gases, this system allows for efficient water recovery with minimal energy input for the pump, which compresses the low-pressure non-condensable gas to atmospheric pressure. Conversely, in Case II, the pump is situated directly downstream of the membrane. Recompression is conducted on the entirety of the downstream flow, which includes both steam and non-condensable gases. This process necessitates a larger energy input compared to Case I. However, the compressed downstream – which predominantly comprises pure steam – can serve as a useful heat medium that condenses at high temperatures, thereby releasing latent heat. Detailed analyses of the water and heat recovery processes within these systems are presented in the remainder of this section.

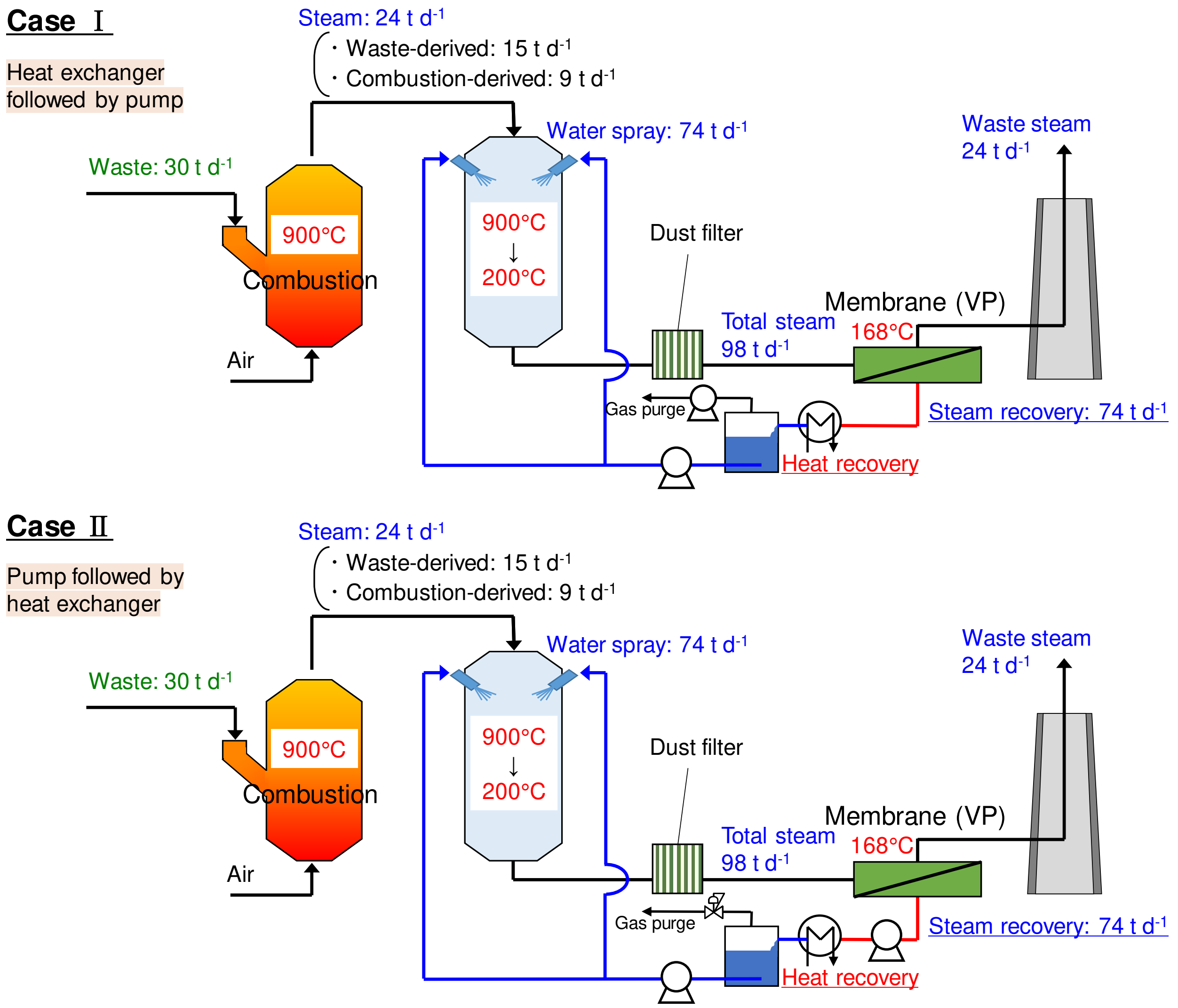

Supplementary Figure 5 Schematic of steam recovery in a waste incinerator plant with a waste capacity of 30 t d^{-1}. (Case Ⅰ: Heat exchanger followed by pump, Case Ⅱ: Pump followed by heat exchanger).

Supplementary Note 3-2: Example of streams in membrane unit and heat exchanger

In our proposed system (Supplementary Figure 6), the steam is condensed downstream from the membrane unit. The membrane recovers steam from the upstream flue gas, and the heat exchanger recovers condensed water and heat. In the membrane unit, the upstream and the downstream flow in opposite directions in a so-called "counter-current", which is the flow pattern that maximizes the membrane unit performance. The heat exchanger is located downstream from the membrane unit where coolant flows counter-currently. This is also referred to as the "counter-current" and is the preferred flow pattern for heat exchangers in industrial applications. In counter-current mode, the flow downstream from the membrane is cooled along the heat exchanger from the inlet to the outlet. The outlet temperature is usually designed to be close to the coolant inlet temperature, typically with a temperature difference of 10-15 °C. On the other hand, the temperature of the coolant increases towards the outlet, which is designed to be close to the temperature of the inlet stream from the membrane. In Case Ⅰ, the coolant evaporates in the first region to maintain the temperature difference between the downstream of the membrane and the coolant, which reduces the amount of coolant. Then, after reaching position-*x*, the coolant completely evaporates. In Case Ⅱ, the coolant remains in the liquid phase throughout the heat exchanger. Supplementary Tables 3 and 4 summarize the assumptions made for the subsequent simulations of membrane separation and the heat exchanger, respectively.

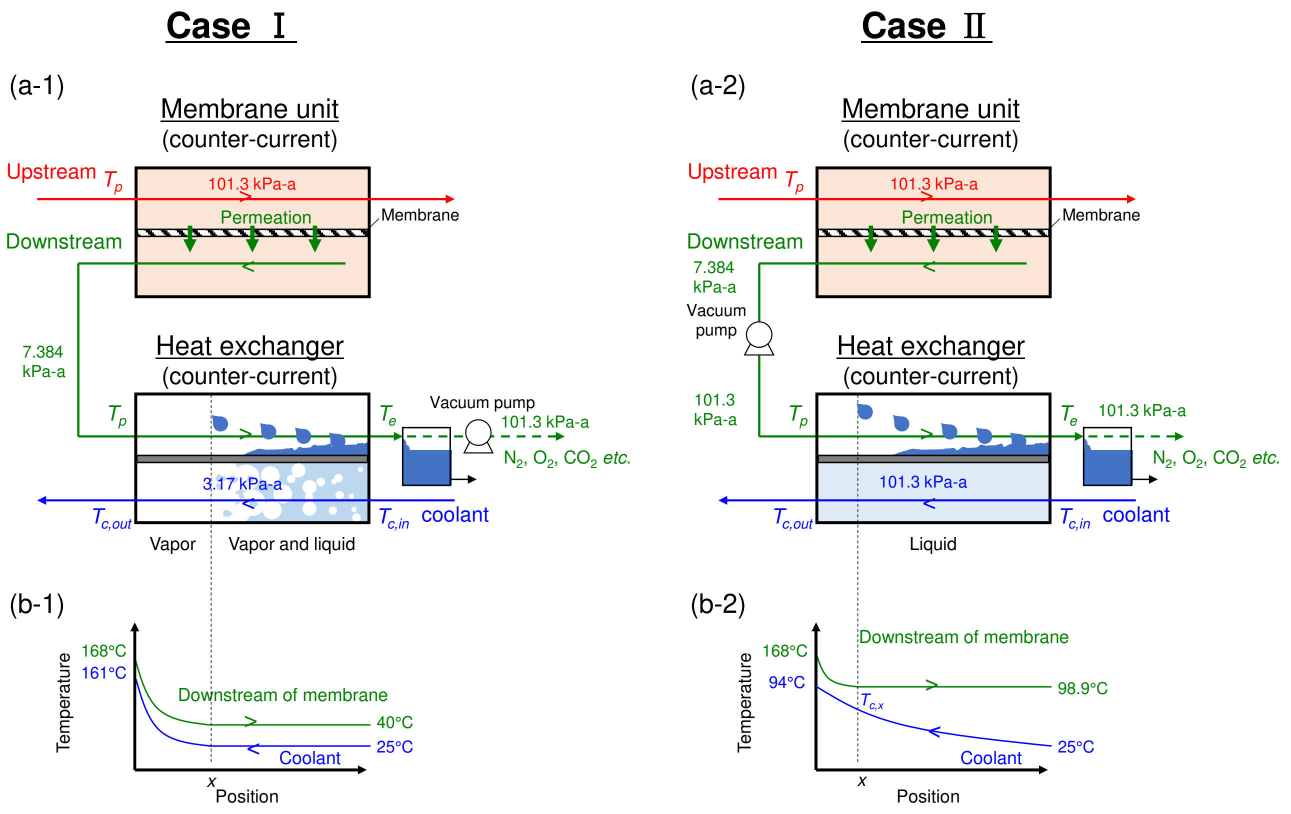

Supplementary Figure 6 Schematic of (a) counter-current flows in the membrane unit and the heat exchanger, and (b) temperatures along the heat exchanger. (a-1) and (b-1) are for Case Ⅰ and (a-2) and (b-2) are for Ⅱ. The temperatures of the downstream and the coolant are constants in the regions where

phase transitions occur. In Case Ⅰ, liquid water at 3.17 kPa-a, which evaporates at 25 °C, is assumed as a simple coolant. In Case Ⅱ, pressurized liquid water is assumed as a coolant.

Supplementary Table 3 Assumptions used for these simulations of membrane separation (for Case Ⅰ and Case Ⅱ); T_p and T_e are the temperatures at the points illustrated in Supplementary Figure 6.

Feed stream			Membrane		
Flow rate [m^3(STP) h^{-1}]	Composition [%]	T_p [°C]	Permeance [mol m^{-2} s^{-1} Pa^{-1}]	Upstream pressure [kPa-a]	Downstream pressure [kPa-a]
13,200 (ref. Table 1)	$H_2O/N_2/O_2/CO_2$=38.9/41.4/10.6/9.1 (ref. Table 1)	168 (ref. Table 1)	H_2O: 2.3×10^{-6}<br>N_2: 1.4×10^{-8}<br>O_2: 3.4×10^{-8}<br>CO_2: 6.5×10^{-8}<br>(ref. Fig 3 (b))	101.3	7.384 (Saturated water vapor pressure at T_e)

Supplementary Table 4 Assumptions used for these simulations of the heat exchanger; T_p, T_e, $T_{c,in}$ and $T_{c,x}$ are the temperatures at the points illustrated in Supplementary Figure 6.

Case	Downstream from membrane			Coolant type			
	T_p [°C]	T_e [°C]	Pressure [kPa-a]	Coolant type	$T_{c,in}$ [°C]	$T_{c,x}$ [°C]	Pressure [kPa-a]
Case Ⅰ	168 (ref. Table 1)	40	7.38 (Saturated water vapor pressure at T_e)	Liquid water	25	25	3.17
Case Ⅱ	168 (ref. Table 1)	(98.9)*	101.3	Liquid water	25	90	101.3

*Calculated from dew point of downstream from the membrane after compression to 101.3 kPa-a.

Supplementary Note 3-3: Example of steam recovery via membrane (for Case Ⅰ and Case Ⅱ)

The assumptions used for this simulation are summarized in Supplementary Table 3. Under the assumption of a counter-current plug flow, the mass balance of the *i*-th component in the upstream is expressed by Eq. (3).

$$-\frac{dF_i}{dA_m} = \Pi_i(p_{u,i} - p_{d,i}) \quad \text{Eq. (3)}$$

In Eq. (3), F_i, $p_{u,i}$, and $p_{d,i}$ indicate the molar flow rate in the upstream, and the partial pressures in both the upstream and downstream of the *i*-th component, respectively, all of which are variable depending on the membrane area, A_m. Π_i is the permeance of the *i*-th component, which was evaluated via the steam recovery experiment.

The mass balance of the *i*-th component in the downstream is expressed by Eq. (4).

$$\frac{dQ_i}{dA_m} = \Pi_i(p_{u,i} - p_{d,i}) \quad \text{Eq. (4)}$$

In Equation (4), Q_i is the molar flow rate of the *i*-th component in the downstream.

The molar flow rate of each component in the upstream and in the downstream along the membrane was integrated under the initial conditions summarized in Table 1 (Main manuscript).

Supplementary Figure 7 shows the permeating flow rates as a function of the membrane area. The Flux of all components increased as the membrane area increased. It is noteworthy that steam had much higher rate of flux than the others. A membrane area of 1,100 m^2 enables steam recovery of 74 t d^{-1}, which realizes a self-reliant waste incinerator plant.

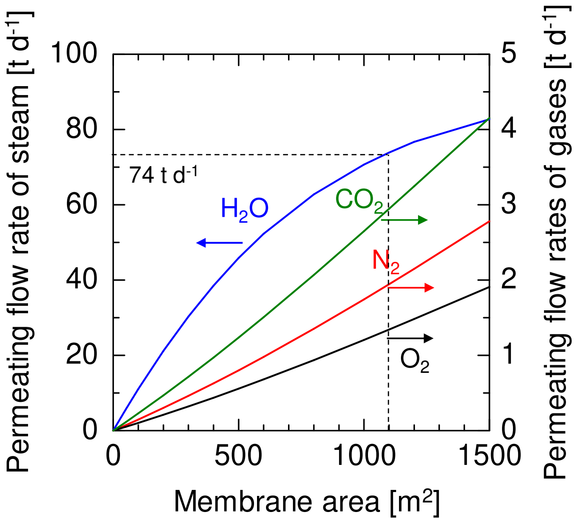

Supplementary Figure 7 Permeating flow rates as a function of the membrane area. For this simulation, the waste stream properties in Table 1 (Main manuscript) and the membrane performance in Fig. 3 (b) (Main manuscript) were used. Of note, the simulation ignored contaminants with a low concentration (ppm level).

Supplementary Note 3-4: Energy recovery via heat exchanger

Supplementary Table 4 summarizes the assumptions for the heat exchanger.

Case Ⅰ

For the sake of simplicity in this simulation, liquid water at 3.17 kPa-a, which evaporates at 25 °C, was used as a coolant. In addition, we simulated the simplest case scenario, which involved the coolant evaporating at the condensation position downstream from the membrane.

The heat removed from downstream from the membrane at the heat exchanger is expressed as Eq. (S5).

$$Heat\ removal = \sum_{i=1}^{4}\left(\int_{T_e}^{T_p} Q_i c_{p,g} dT\right) + Lv_{T_e} Q_s \quad \text{Eq. (S5)}$$

In Eq. (S5), Q_s, and Q_i indicate the permeating flow rates [mol s^{-1}] of steam and the *i*-th components, respectively. T_p and T_e are the temperatures [K] of the process waste stream and the permeate stream following the use of a heat exchanger, respectively. Lv_{T_e} and $c_{p,g}$ are the latent heat of water at T_e [J mol^{-1}] and the heat capacity of gas as a function of temperature [J mol^{-1} K^{-1}], respectively.

In addition, the heat obtained by the coolant, which corresponds to the heat removal in Eq. (5), is expressed as Eq. (S6).

$$Obtained\ heat = Q_c \int_{T_{c,in}}^{T_{c,out}} c_{p,c} dT + Lv_{T_{c,in}} Q_c \quad \text{Eq. (S6)}$$

In Eq. (S6), Q_c, $T_{c,in}$ and $T_{c,out}$, and $c_{p,c}$ are the flow rate [mol s^{-1}], temperatures at inlet and outlet [K], and the heat capacity of the coolant as a function of temperature [J mol^{-1} K^{-1}], respectively.

For the simplest example, we estimated coolant evaporation at the position where condensation occurred downstream from the membrane. Thus, Eqs. (S5) and (S6) give Eqs. (S7) and (S8).

$$Lv_{T_e} Q_s = Lv_{T_{c,in}} Q_c \quad \text{Eq. (S7)}$$

Here, the thermodynamic parameters are found in SI-10. In Eq. (S7), when Q_s=74 t d^{-1}, a self-reliant waste incinerator plant is realized; T_e=40 °C and $T_{c,in}$=25 °C, and, therefore, $\underline{Q_c\text{=72.9 t d}^{-1}}$.

$$\sum_{i=1}^{4}\left(\int_{T_e}^{T_p} Q_i c_{p,g} dT\right) = Q_c \int_{T_{c,in}}^{T_{c,out}} c_{p,c} dT \quad \text{Eq. (S8)}$$

The membrane area needed to recover 74 t d^{-1} of steam is 1,100 m^2, as shown Supplementary Figure 7 and Fig. 5 (a) (Main manuscript), and the flow rates for each of the components in the downstream, Q_i, appear in Supplementary Figure 7. T_p=168 °C (average temperature in Table 1 (Main manuscript)), T_e=40 °C, Q_c=72.9 t d^{-1}, and $T_{c,in}$=25 °C, which results in $\underline{T_{c,out}\text{=161 °C}}$.

When considering heat transfer via a heat exchanger, Eq. (S9) is used.

$$Transfered\ heat = U_v A_{h,v} \frac{(T_p - T_{c,out}) - (T_e - T_{c,in})}{ln(T_p - T_{c,out}) - ln\ (T_e - T_{c,in})} + U_{v/l} A_{h,v/l}(T_e - T_{c,in}) \quad \text{Eq. (S9)}$$

In Eq. (S9), U_v and $A_{h,v}$, respectively, are the overall heat transfer coefficient and the area of the heat exchanger in the vapor region, and $U_{v/l}$ and $A_{h,v/l}$, respectively, are those in the vapor/liquid phase transition region.

On the right side of Eq. (S9), the first and second terms indicate transferred heat in the vapor and vapor/liquid phase transition regions, respectively. Therefore,

$$U_v A_{h,v} \frac{(T_p - T_{c,out}) - (T_e - T_{c,in})}{ln(T_p - T_{c,out}) - ln\,(T_e - T_{c,in})} = \sum_{i=1}^{4} \left(\int_{T_e}^{T_p} Q_i c_{p,g} dT \right) \quad \text{Eq. (S10)}$$

$$U_{v/l} A_{h,v/l} (T_e - T_{c,in}) = Lv_{T_e} Q_s \quad \text{Eq. (S11)}$$

Eqs. (S10) and (S11) provide $U_v A_{h,v}$=20.1 kW K^{-1} and $U_{v/l} A_{h,v/l}$=137 kW K^{-1}, respectively.

Supplementary Table 5 summarizes an example of a construct of a membrane unit and a heat exchanger with the result of the simulation of Case Ⅰ. A membrane area of 1,100 m^2 is needed to recover 74 t d^{-1} of steam at 168 °C, which realizes a self-reliant waste incinerator plant. Energy from the latent and sensitive heat of the recovered steam reached quantities as large as 195 GJ d^{-1} when using a heat exchanger with a capacity of $U_v A_{h,v}$ 20.1 kW K^{-1} and $U_{v/l} A_{h,v/l}$=137 kW K^{-1}, using 72.9 t d^{-1} of coolant liquid water at 25 °C. That setup resulted in 72.9 t d^{-1} of water vapor at 161 °C.

Supplementary Table 5 An example of the construction of a simulated membrane and heat exchanger with coolant liquid water at 3.17 kPa-a (Case Ⅰ).

Membrane			Heat exchanger						
Membrane area [m^2]	T_p [°C]	Steam recovery [t d^{-1}]	Heat recovery [GJ d^{-1}]	$U_v A_{h,v}$ [kW K^{-1}]	$U_{v/l} A_{h,v/l}$ [kW K^{-1}]	Flow rate of coolant [t d^{-1}]	$T_{c,in}$ [°C]	$T_{c,out}$ [°C]	T_e [°C]
1,100	168	74	195	20.1	137	72.9	25	161	40

Case Ⅱ

According to Supplementary Figure 7, with the membrane area of 1,100 m^2, the composition of steam in downstream from the membrane is 95.8%. In the simulation, this stream is assumed to be recompressed to 101.3 kPa-a prior to entering to the heat exchanger. Therefore, the water vapor pressure at the inlet of the heat exchanger is 101.3×0.958 = 97.0 kPa-a, which corresponds to the dew point of 98.9°C. Therefore, T_e=98.9 °C is given for Case Ⅱ. It should be noted that T_e can be controlled by tunning the downstream pressure at the inlet of the heat exchanger. The maximum is 168°C, which is the temperature at the inlet of the heat exchanger and the membrane unit.

Similar to Case I, the heat removed downstream from the membrane and the heat obtained are expressed as Eqs. (S12) and (S13).

$$Heat\ removal = \sum_{i=1}^{4}\left(\int_{T_e}^{T_p} Q_i c_{p,g} dT\right) + Lv_{T_e} Q_s \quad \text{Eq. (S12)}$$

$$Obtained\ heat = Q_c \int_{T_{c,x}}^{T_{c,out}} c_{p,c} dT + Q_c \int_{T_{c,in}}^{T_{c,x}} c_{p,c} dT \quad \text{Eq. (S13)}$$

In Eq. (S13), $T_{c,x}$ is the coolant temperature at the position-*x* (in Supplementary Figure 6).
Considerations of the heat balances in regions where the downstream from the membrane is in the vapor phase and in vapor/liquid transition provide Eqs. (S14) and (S15), respectively.

$$Lv_{T_e} Q_s = Q_c \int_{T_{c,in}}^{T_{c,x}} c_{p,c} dT \quad \text{Eq. (S14)}$$

$$\sum_{i=1}^{4}\left(\int_{T_e}^{T_p} Q_i c_{p,g} dT\right) = Q_c \int_{T_{c,x}}^{T_{c,out}} c_{p,c} dT \quad \text{Eq. (S15)}$$

When $T_{c,x}$ of 90 °C is assumed, Eq. (S14) gives Q_c=613 t d^{-1}. Subsequently, Eq. (S15) gives $T_{c,out}$=94 °C.

When considering heat transfer through a heat exchanger, Eq. (S16) is used.

$$Transfered\ heat = U_v A_{h,v} \frac{(T_p - T_{c,out}) - (T_e - T_{c,x})}{ln(T_p - T_{c,out}) - \ln(T_e - T_{c,x})} + U_{v/l} A_{h,v/l} \frac{(T_e - T_{c,x}) - (T_e - T_{c,in})}{ln(T_e - T_{c,x}) - \ln(T_e - T_{c,in})} \quad \text{Eq. (S16)}$$

As with Case Ⅰ,

$$U_v A_{h,v} \frac{(T_p - T_{c,out}) - (T_e - T_{c,x})}{ln(T_p - T_{c,out}) - \ln(T_e - T_{c,x})} = \sum_{i=1}^{4}\left(\int_{T_e}^{T_p} Q_i c_{p,g} dT\right) \quad \text{Eq. (S17)}$$

$$U_{v/l} A_{h,v/l} \frac{(T_e - T_{c,x}) - (T_e - T_{c,in})}{ln(T_e - T_{c,x}) - \ln(T_e - T_{c,in})} = Lv_{T_e} Q_s \quad \text{Eq. (S18)}$$

Eqs. (S17) and (S18) provide $U_v A_{h,v}$=3.83 kW K^{-1} and $U_{v/l} A_{h,v/l}$=62.9 kW K^{-1}, respectively.

Supplementary Table 6 summarizes an example of a construction of a membrane unit and a heat exchanger based on the result of the simulation for Case Ⅱ. The membrane unit, which has 1,100 m^2 of membrane area and recovers 74 t d^{-1} of steam at 168 °C, is the same with Case Ⅰ. In Case Ⅱ, energy from the latent and sensitive heat of the recovered steam reached quantities as large as 177 GJ d^{-1} when using a heat exchanger with a capacity of $U_v A_{h,v}$ 3.8 kW K^{-1} and $U_{v/l} A_{h,v/l}$=62.9 kW K^{-1}. This setup produces 613 t d^{-1} of hot water at 161 °C.

Supplementary Table 6 An example of the construction of a simulated membrane and heat exchanger with coolant liquid water at 101.3 kPa-a (Case II).

Membrane			Heat exchanger						
Membrane area [m^2]	T_p [°C]	Steam recovery [t d^{-1}]	Heat recovery [GJ d^{-1}]	$U_v A_{h,v}$ [kW K^{-1}]	$U_{v/l} A_{h,v/l}$ [kW K^{-1}]	Flow rate of coolant [t d^{-1}]	$T_{c,in}$ [°C]	$T_{c,out}$ [°C]	T_e [°C]
1,100	168	74	177	3.8	62.9	613	25	94	99

Supplementary Note 3-5: Recovery and operational energy costs

Supplementary Figure 8 shows the quantities of recovered steam, along with the recovered and consumed energy, based on the above simulation. Additionally, the cost of the recovered steam and energy consumed by the vacuum pump were calculated based on the cost to produce steam[22] and the cost of green electricity[23], since the heat recovery and energy consumed by the vacuum pump cannot be directly compared. Here, we assumed the construction of the membrane unit as summarized in Supplementary Tables 5 (for Case I) and 6 (for Case II). The compression energy for vacuum pumps was calculated via Eq. (S19) under assumption of isothermal compression with ideal compression efficiency.

$$W = \int_{P_{in}}^{P_{out}} \frac{QRT}{P} dP \quad (S19)$$

In Eq. (S19), W, P_{out}, P_{in}, Q, and T are energy consumption, pressures at the outlet and the inlet of the pump, molar flow rate, and temperature, respectively.

Both Case I and Case II achieved steam recovery of 74 t d^{-1}, which enables the self-reliant operation of an incinerate plant. The energy recovery in Case I and Case II were, respectively, more than 100-fold and 5-fold the energy consumption of a vacuum pump, suggesting the effectiveness of these steam recovery systems not only for water recovery, but also for energy recovery. More importantly, the value of the recovered steam exceeded the cost of running a vacuum pump by a factor of eight to several hundreds. Thus, the effectiveness of the proposed system can also be assessed from an economic perspective.

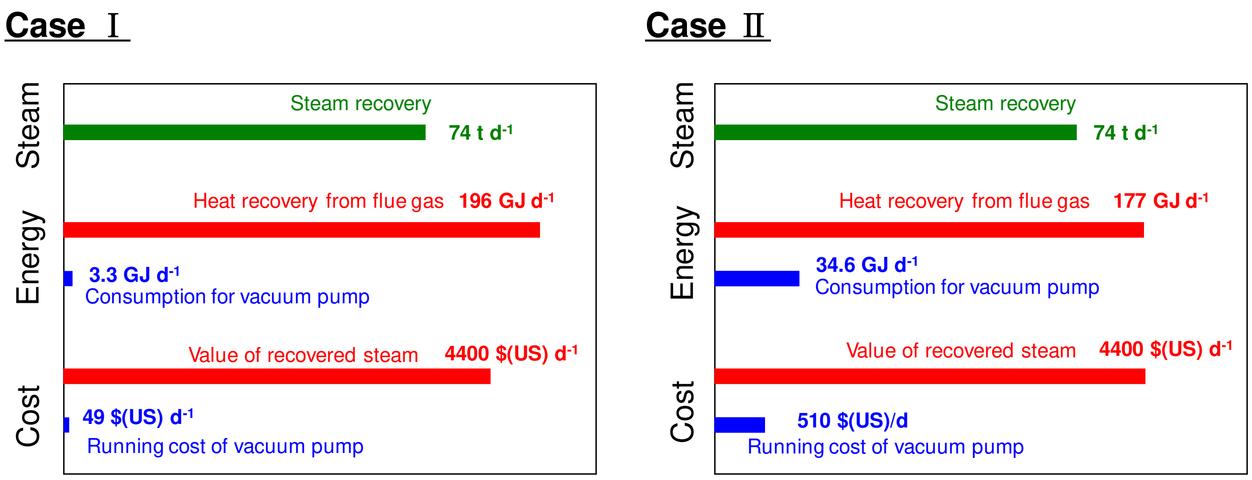

Supplementary Figure 8 Evaluation of the proposed system: The value of heat recovery via a heat exchanger after a membrane unit and running energy for vacuum pump with their costs. Here, the conditions summarized in Supplementary Table 3 were assumed. The energies were estimated according to Eq. (S19). The costs to produce steam (60 $(US) t^{-1}) [22] and green electricity (0.053 $(US) kWh^{-1}) [23] were used to estimate the recovered and consumed costs, respectively.

Supplementary Note 4: Competing technologies for steam recovery

Competing technologies used for steam recovery are listed in Supplementary Figure 9. A heat exchanger is the simplest component of the equipment because it simply cools the entire stream and traps liquified water. However, the trapped water will contain contaminants. Table 1 (Main manuscript) summarizes the stream properties after passage through a dust filter during the operation of a waste incinerator plant in Japan. When the trapped water is reused, acidic components such as HCl, NO_x and SO_x are dissolved in the trapped water and must be concentrated, but this tends to accelerate corrosion in the heat exchangers and pipes. The existence of non-condensable gases reduces the heat-transfer coefficient by approximately half. In addition, the retentate stream, which is the outlet stream of the feed from the heat exchanger, is saturated, and this produces the appearance of a steam condensate plume from the stack. A transport membrane condenser (TMC) uses nanoporous membranes (pore size: several to several tens nm) to achieve separation where the steam is fed to the feed side and cold water is fed to the downstream. [24-27] Capillary forces generated in the membrane pores allow the condensation and permeation of water and water-soluble components but prevents that of others. Wang *et al.* demonstrated steam recovery via a TMC in a power plant and reported an improvement in energy efficiency of >5%.[27] However, a TMC requires water condensation, and we could find no reports of the use of a TMC at temperatures higher than 120 °C. [14] Therefore, use of a TMC would be difficult for steam recovery in waste incinerator plants that typically are operated at 150-170 °C. In addition, the retentate stream from a TMC is cooled in an almost saturated state, so that the formation of a steam condensate plume from the stacks is inevitable. Vapor permeation (VP), which we proposed for this new system, is a strategy where conditions of both the upstream and downstream of the membrane are in the vapor phase. These membranes have non-porous or subnanoporous structures, enabling almost pure steam recovery in the downstream. [28] Moreover, the recovery of latent heat via a heat exchanger in the downstream is a significant development. Since the mole fraction in the downstream of steam is approximately 100%, the heat transfer efficiency should be high because of negligible non-condensable components, as illustrated in Supplementary Figure 2. In addition, as noted above, the retentate stream can be dehumidified without decreasing the temperature to prevent the appearance of a steam condensate plume from stacks. Therefore, VP is the most suitable strategy for this system.

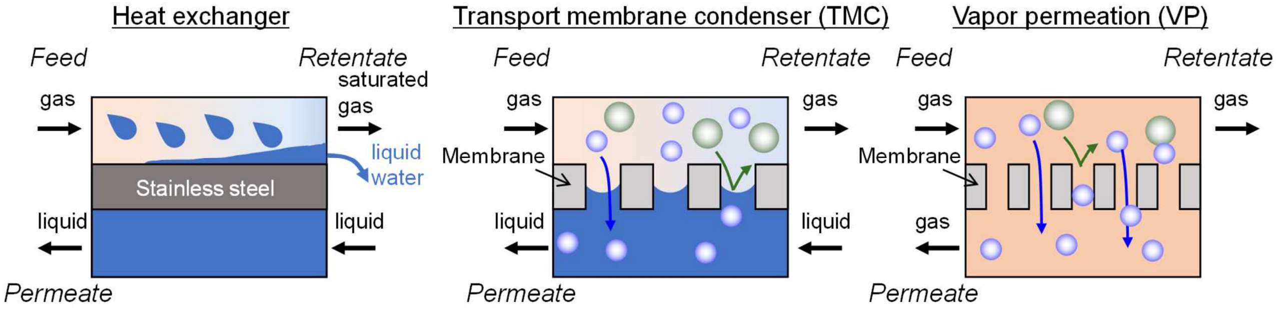

Supplementary Figure 9 Competing technologies for steam recovery.

Supplementary Note 5: Membrane performance after long-term stability testing (laboratory-scale test)

Supplementary Table 7 Compositions of the feed and permeate downstream following 190 days in a steam-recovery test (Fig. 2 (c), main manuscript)

	H_2O [mol%]	HCl [mol%]	N_2 [mol%]
Feed	50.00	4.0×10^{-3}	50.00
Permeate (downstream)	99.34	0.30×10^{-3}	0.66

Supplementary Figure 10 compares the steam/nitrogen separation performances of BTESE-derived organosilica membranes with those of other membranes. Generally, BTESE-organosilica membranes show high steam permeance with a sufficient level of permeance ratios. The performance of M-2 in a 190-day steam recovery test (Fig. 2 (c), main manuscript) is comparable to BTESE-organosilica membranes described in the literature, [11,13,14] which confirms the high levels of hydrothermal, oxidative and acidic stability of organosilica membranes as well as high levels of steam/nitrogen separation performance.

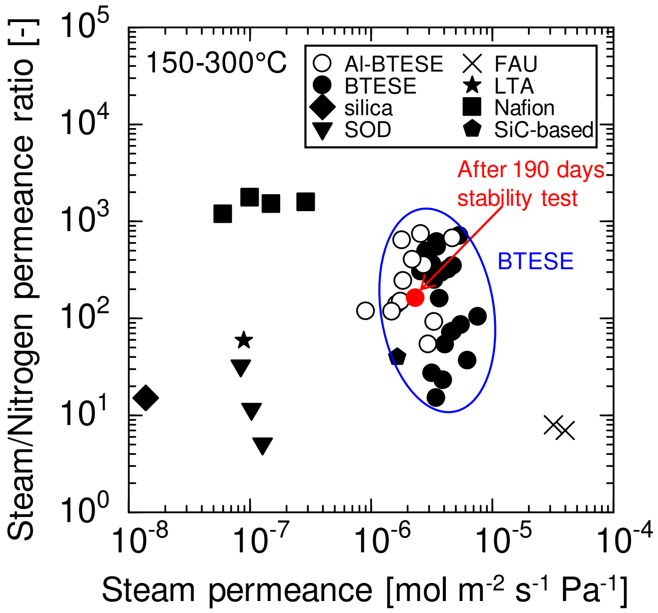

Supplementary Figure 10 A summary of characterized membrane performance in the literature: The relationship between steam permeance and a steam/nitrogen permeance ratio at 150-300 °C. The permeance is used for comparison instead of permeability, since permeance directly reflects the actual performance of as-prepared membranes from each material. The original data can be found elsewhere. [7, 11, 13, 14, 21, 29-32] Source data are provided as a Source Data file.

Supplementary Note 6: Time course of steam recovery experiments

Supplementary Figure 11 shows the time courses for the permeance of steam, hydrogen chloride, and nitrogen through the M-2. In this experiment, streams with different HCl concentrations (0-400 ppm) were fed periodically to the membrane while maintaining a steam composition, x, at 0.5. Even though the membrane was exposed to a HCl concentration that reached 400 ppm at its maximum, the values for steam and nitrogen permeance evaluated at the initial and at the end of this experiment without HCl remained almost constant, which confirmed the excellent acidic stability of this membrane and the reproducibility of the experimental results.

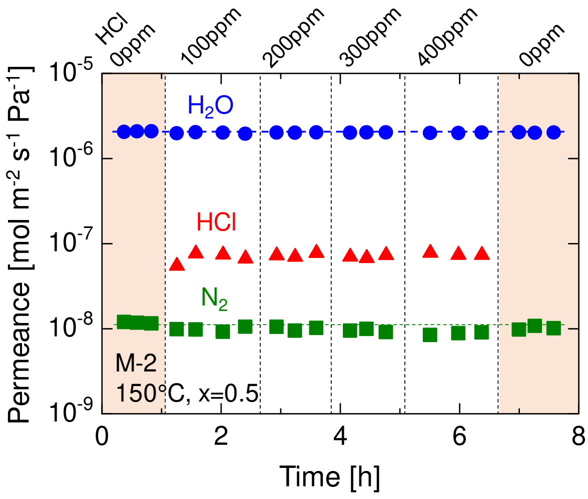

Supplementary Figure 11 Time courses for permeance during steam recovery with different concentrations of HCl. This experiment was performed on day 112 (as shown in Fig. 2 (c)). Source data are provided as a Source Data file.

Supplementary Figure 12 shows the time course of steam recovery performance from equimolar binary mixtures of steam and various types of gases at 150 °C. It should be noted that, exceptionally, $H_2O/HCl/N_2$ ternary separation was performed on an equimolar mixture of steam and nitrogen with 40 ppm of hydrogen chloride. The permeance and permeance ratio of H_2O/N_2 binary separation were periodically measured and showed stable values, which confirms the reproducibility of the measurement.

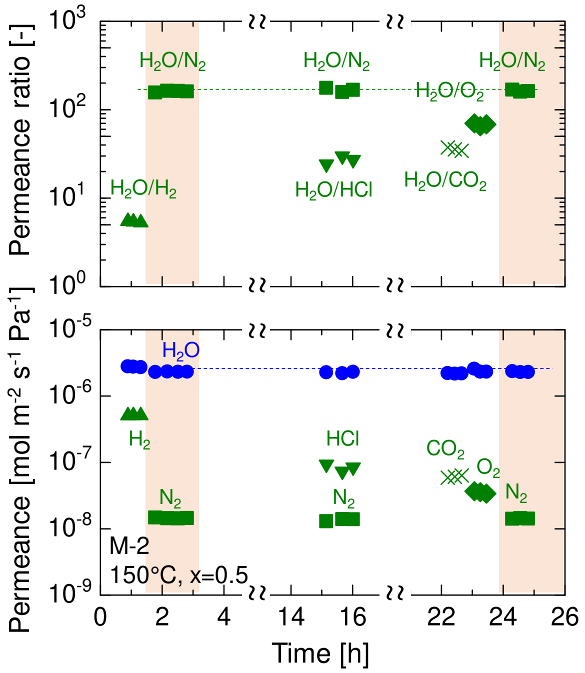

Supplementary Figure 12 Time course of steam recovery performance from several steam/gas mixtures at 150 °C with the steam mole fraction in the feed stream maintained at 0.5. This experiment was performed on days 190 and 191 (Fig. 2 (c)). Source data are provided as a Source Data file.

Supplementary Note 7: Downstream pressures in the bench-scale apparatus

Supplementary Figure 13 illustrates the ideal pressure distribution of the process flow downstream from the membrane unit in a bench-scale test. The temperatures in this figure refer to the values in Fig. 4 (a) (Main manuscript). When the capacity of the vacuum pump is adequate to completely remove permeating non-condensable gases (in an ideal case), the total pressure on the downstream side of the membrane module can be reduced to the point of saturated water vapor pressure at the lowest temperature along the heat exchanger. However, in this bench-scale test where the capacity of the vacuum pump was insufficient, the total pressure on the downstream side of the membrane module increased to balance the permeating flow rate with the evacuating flow rate. The composition of component-*i* in the downstream is equivalent to the value of the ratio of the flux of component-*i* to the total flux. Along the heat exchanger, water vapor pressure decreases down to the saturated pressure at the lowest temperature of the heat exchanger, and the partial pressure of non-condensable gases increases due to accumulation.

In the present study, the vacuum pump capacity was sufficient for the laboratory-scale tests but appeared to be insufficient for the bench-scale test. For industrial applications, a significantly larger vacuum pump capacity would be desirable. Therefore, the pressure prior to vacuum pumping (Fig. 4 (a), Main manuscript) was 40 kPa-a, which was larger than 2.3 kPa-a: the saturated water vapor pressure at 20°C. However, the results from the bench-scale test affirmed and confirmed the concept of the proposed system. The water permeance — which is the water flux divided by the partial pressure difference across the membrane — remained consistent in both the laboratory-scale test and the bench-scale test (Fig. 4 (b)). This consistency confirmed the desirable steam recovery performance of the membrane unit, even from the actual waste stream of a running incinerator plant.

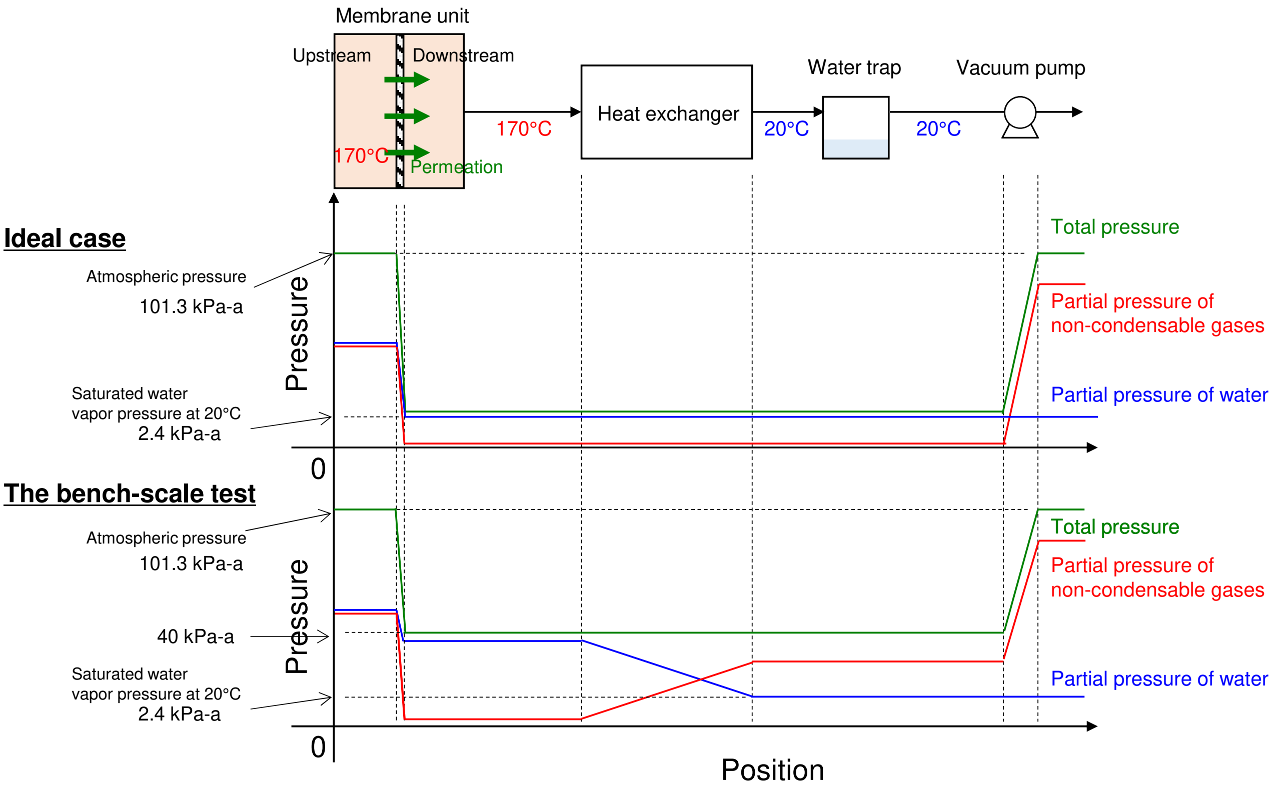

Supplementary Figure 13 Schematics of the downstream pressure distribution for both sufficient and insufficient evacuation. Here, the pressure drop in the process pipes is neglected.

Supplementary Note 8: Cross-sectional SEM image of an organosilica membrane

Supplementary Figure 14 presents a cross-sectional SEM image of a BTESE-derived organosilica membrane. The layer composed of particles sized at 200 nm is the surface of an α-alumina support smoothed by coating a mixture of α-alumina particles and BTESE-swing sols. The layer composed of 10-40 nm particles is a BTESE-swing sols-derived intermediate with a thickness of 150-200 nm. The top dense layer coated on the intermediate layer is a BTESE-acid sols-derived separation layer which is effective for separation. The separation layer was as thin as 40 nm, and no cracks were observed. In this study, for the first time, we have confirmed the excellent hydrothermal stability (150-200°C, 50 kPa-H_2O, HCl: 40 ppm, over 6 months) of an organosilica membrane with the layered structure where the separation layer consists of molecular sieving organosilica network.

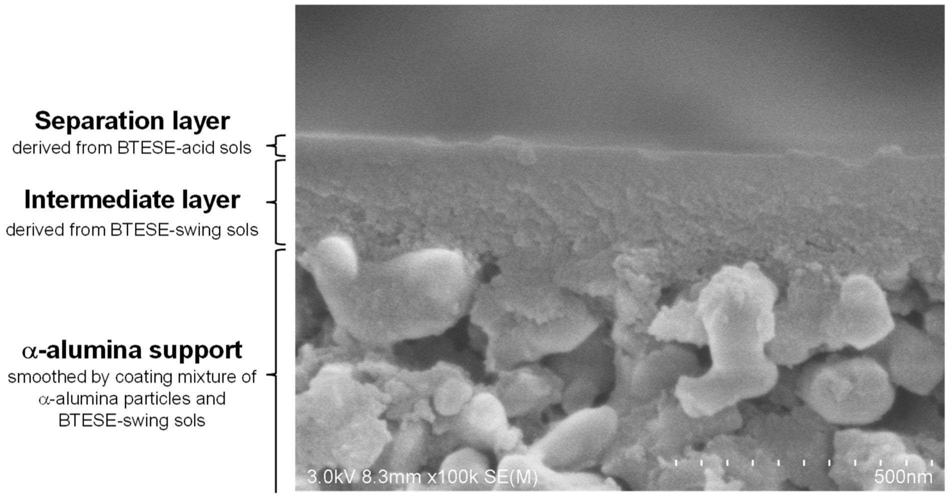

Supplementary Figure 14 A cross-sectional SEM image of a BTESE-derived organosilica membrane.

Supplementary Note 9: Thermodynamic properties used to evaluate effective heat recovery

Supplementary Table 8 Latent heat of water[33, 34]

Temperature [°C]	Latent heat [kJ kg^{-1}]
10	2477.7
25	2442.3
40	2406.7
98.9	2258.7

Heat capacity of gas, $c_{p,g}$, as a function of temperature was estimated using Eq. (S20).

$$\frac{c_{p,g}}{R} = \mathrm{a0} + \mathrm{a1}T + \mathrm{a2}T^2 + \mathrm{a3}T^3 + \mathrm{a4}T^4 \quad \mathrm{Eq.}\,(S20)$$

In Eq. (S19), R is the gas constant [J mol^{-1} K^{-1}] and T is the temperature [K]

On the other hand, heat capacity of liquid, $c_{p,l}$, as a function of temperature was estimated using Eq. (S21).

$$c_{p,g} = \mathrm{a0} + \mathrm{a1}T + \mathrm{a2}T^2 + \mathrm{a3}T^3 + \mathrm{a4}T^{-2} \quad \mathrm{Eq.}\,(S21)$$

Supplementary Table 9 Constants of a0, a1, a2, a3, and a4 in Eq. (S20) [35] and in Eq. (S21)[36].

Component	a0	a1×10^3	a2×10^5	a3×10^8	a4×10^{11}
H_2O (gas)	4.395	-4.186	1.405	-1.564	0.632
N_2	3.539	-0.261	0.007	0.157	-0.099
O_2	3.630	-1.794	0.658	-0.601	0.179
CO_2	3.259	1.356	1.502	-2.374	1.056
H_2O (liquid)	-203.606	1523.29	-3196.413	2474.455	3.855326×10^{17}

Saturated water vapor pressures for all temperatures and dew points were estimated using Eq. (S22).

$$\log_{10} p_s = \mathrm{A} - \frac{\mathrm{B}}{T + \mathrm{C}} \quad \mathrm{Eq.}\,(S22)$$

In Eq. (S17), p_s [bar] and T [°C] are the vapor pressure and temperature, respectively. A, B, and C are the Antoine coefficients, which correspond to 5.11564; 1,687.537; and, 230.17, respectively. [35]

Supplementary References

1 Slegers, L. & Sebar, R. A. Laminar film condensation of steam containing small concentrations of airFilm laminaire de condensation d'une vapeur contenant de l'eau en faible concentrationLaminare film-kondensation von dampf mit geringem luftgehalt. *Int. J. Heat Mass Transfer* **13**, 1941-1947 (1970).

2 Bolto, B., Hoang, M. & Xie, Z. A review of water recovery by vapour permeation through membranes. *Water Res.* **46**, 259-266 (2012). https://doi.org:10.1016/j.watres.2011.10.052

3 Liang, C. Z. & Chung, T.-S. Robust thin film composite PDMS/PAN hollow fiber membranes for water vapor removal from humid air and gases. *Sep. Purif. Technol.* **202**, 345-356 (2018). https://doi.org:10.1016/j.seppur.2018.03.005

4 Qu, M., Abdelaziz, O., Gao, Z. & Yin, H. Isothermal membrane-based air dehumidification: A comprehensive review. *Renewable and Sustainable Energy Reviews* **82**, 4060-4069 (2018). https://doi.org:10.1016/j.rser.2017.10.067

5 Xing, R. *et al.* Advanced thin zeolite/metal flat sheet membrane for energy efficient air dehumidification and conditioning. *Chem. Eng. Sci.* **104**, 596-609 (2013). https://doi.org:10.1016/j.ces.2013.08.061

6 Li, G. M., Feng, C., Li, J. F., Liu, J. Z. & Wu, Y. L. Water vapor permeation and compressed air dehydration performances of modified polyimide membrane. *Sep. Purif. Technol.* **60**, 330-334 (2008). https://doi.org:10.1016/j.seppur.2007.05.007

7 Azher, H., Scholes, C. A., Stevens, G. W. & Kentish, S. E. Water permeation and sorption properties of Nafion 115 at elevated temperatures. *J. Membr. Sci.* **459**, 104-113 (2014). https://doi.org:10.1016/j.memsci.2014.01.049

8 Rohde, M. P., Schaub, G., Khajavi, S., Jansen, J. C. & Kapteijn, F. Fischer–Tropsch synthesis with in situ H2O removal – Directions of membrane development. *Microporous Mesoporous Mater.* **115**, 123-136 (2008). https://doi.org:10.1016/j.micromeso.2007.10.052

9 van Kampen, J., Boon, J., van Berkel, F., Vente, J. & van Sint Annaland, M. Steam separation enhanced reactions: Review and outlook. *Chem. Eng. J.* **374**, 1286-1303 (2019). https://doi.org:10.1016/j.cej.2019.06.031

10 Metz, S., van de Ven, W., Potreck, J., Mulder, M. & Wessling, M. Transport of water vapor and inert gas mixtures through highly selective and highly permeable polymer membranes. *J. Membr. Sci.* **251**, 29-41 (2005). https://doi.org:10.1016/j.memsci.2004.08.036

11 Moriyama, N., Ike, M., Nagasawa, H., Kanezashi, M. & Tsuru, T. Network tailoring of organosilica membranes via aluminum doping to improve the humid-gas separation performance. *RSC Adv.* **12**, 5834-5846 (2022). https://doi.org:10.1039/d1ra07866f

12 Moriyama, N., Nagasawa, H., Kanezashi, M. & Tsuru, T. Selective water vapor permeation from steam/non-condensable gas mixtures via organosilica membranes at moderate-to-high temperatures. *J. Membr. Sci.* **589**, 117254 (2019). https://doi.org:10.1016/j.memsci.2019.117254

13 Moriyama, N., Nagasawa, H., Kanezashi, M. & Tsuru, T. Improved performance of organosilica membranes for steam recovery at moderate-to-high temperatures via the use of a hydrothermally stable intermediate layer. *J. Membr. Sci.* **620**, 118895 (2021). https://doi.org:10.1016/j.memsci.2020.118895

14 Moriyama, N., Nagasawa, H., Kanezashi, M. & Tsuru, T. Steam recovery via nanoporous and subnanoporous organosilica membranes: The effects of pore structure and operating conditions. *Sep. Purif. Technol.* **275**, 119191 (2021). https://doi.org:10.1016/j.seppur.2021.119191

15 Piera, E., Salomón, M. A., Coronas, J., Menéndez, M. & Santamaría, J. Synthesis, characterization and separation properties of a composite mordenite/ZSM-5/chabazite hydrophilic membrane. *J. Membr. Sci.* **149**, 99-114 (1998).

16 Scholes, C. A., Stevens, G. W. & Kentish, S. E. Membrane gas separation applications in natural gas processing. *Fuel* **96**, 15-

28 (2012). https://doi.org:10.1016/j.fuel.2011.12.074

17 Shin, Y. *et al.* Graphene oxide membranes with high permeability and selectivity for dehumidification of air. *Carbon* **106**, 164-170 (2016). https://doi.org:10.1016/j.carbon.2016.05.023

18 Sijbesma, H. *et al.* Flue gas dehydration using polymer membranes. *J. Membr. Sci.* **313**, 263-276 (2008). https://doi.org:10.1016/j.memsci.2008.01.024

19 Suzuki, S., Shoji, N. & Tsuru, T. Performance evaluation of water vapor permeation through perfluorosulfonic acid capillary membranes. *Sep. Purif. Technol.* **266**, 118508 (2021). https://doi.org:10.1016/j.seppur.2021.118508

20 Suzuki, S. & Tsuru, T. Analysis and prediction of water vapor permeation through perfluorosulfonic acid membranes via the solution-diffusion model in a single-membrane dehumidifier module. *Sep. Purif. Technol.* **279**, 119694 (2021). https://doi.org:10.1016/j.seppur.2021.119694

21 Tsuru, T. *et al.* Permeation properties of hydrogen and water vapor through porous silica membranes at high temperatures. *AIChE J.* **57**, 618-629 (2011). https://doi.org:10.1002/aic.12298

22 Mabrouk, A.-N. A., Nafey, A. S. & Fath, H. E. S. Steam, electricity and water costs evaluation of power desalination co-generation plants. *Desalination and Water Treatment* **22**, 56-64 (2012). https://doi.org:10.5004/dwt.2010.1537

23 Renewable power generation costs in 2019. (International Renewable Energy Agency, Abu Dhabi, 2020).

24 Chen, H. *et al.* Heat exchange and water recovery experiments of flue gas with using nanoporous ceramic membranes. *Appl. Therm. Eng.* **110**, 686-694 (2017). https://doi.org:10.1016/j.applthermaleng.2016.08.191

25 Wang, D., Bao, A., Kunc, W. & Liss, W. Coal power plant flue gas waste heat and water recovery. *Applied Energy* **91**, 341-348 (2012). https://doi.org:10.1016/j.apenergy.2011.10.003

26 Wang, T., Yue, M., Qi, H., Feron, P. H. M. & Zhao, S. Transport membrane condenser for water and heat recovery from gaseous streams: Performance evaluation. *J. Membr. Sci.* **484**, 10-17 (2015). https://doi.org:10.1016/j.memsci.2015.03.007

27 Zhao, S. *et al.* Simultaneous heat and water recovery from flue gas by membrane condensation: Experimental investigation. *Appl. Therm. Eng.* **113**, 843-850 (2017). https://doi.org:10.1016/j.applthermaleng.2016.11.101

28 Vane, L. M. Review: Membrane Materials for the Removal of Water from Industrial Solvents by Pervaporation and Vapor Permeation. *J. Chem. Technol. Biotechnol.* **94**, 343-365 (2019). https://doi.org:10.1002/jctb.5839

29 Lafleur, M. *et al.* Development of a water-selective zeolite composite membrane by a new pore-plugging technique. *Microporous Mesoporous Mater.* **237**, 49-59 (2017). https://doi.org:10.1016/j.micromeso.2016.09.004

30 Mastropietro, T. F. *et al.* Study of the separation properties of FAU membranes constituted by hierarchically assembled nanozeolites. *Sep. Purif. Technol.* **156**, 321-327 (2015). https://doi.org:10.1016/j.seppur.2015.10.018

31 Aoki, K., Kusakabe, K. & Morooka, S. Separation of gases with an A-type zeolite membrane. *Ind. Eng. Chem. Res.* **39**, 2245-2251 (2000).

32 Wang, Q., Yu, L., Nagasawa, H., Kanezashi, M. & Tsuru, T. High‐performance molecular‐separation ceramic membranes derived from oxidative cross‐linked polytitanocarbosilane. *Journal of the Am. Ceram. Soc.* **103**, 4473-4488 (2020). https://doi.org:10.1111/jace.17108

33 Keenan, J. H., Keyes, F. G., Hill, P. G. & Moore, J. G. *Steam Tables*. (Wiley, 1969).

34 NIST Chemistry WebBook, Thermophysical Properties of Fluid System, Standard refference database number 69

35 Poling, B. E., Prausnitz, J. M. & O'Connell, J. P. *The properties of gases and liquids*. 5 edn, (McGraw-Hill companies, Inc., 2001).

36 Chase, M. NIST-JANAF Thermochemical Tables, 4 edn. (American Institute of Physics, -1, 1998).
